# Syntactic Networks of Interlanguage Across L2 Modalities and Proficiency Levels

**DOI:** 10.3389/fpsyg.2021.643120

**Published:** 2021-07-14

**Authors:** Yuxin Hao, Xuelin Wang, Meng Wu, Haitao Liu

**Affiliations:** ^1^Institute of Chinese Language and Culture Education, Huaqiao University, Xiamen, China; ^2^Centre for Linguistics and Applied Linguistics, Guangdong University of Foreign Studies, Guangzhou, China; ^3^Department of Linguistics, Zhejiang University, Hangzhou, China

**Keywords:** syntactic networks, interlanguage, dependency syntax, modalities, L2 proficiency

## Abstract

Over time, interlanguage studies have shifted from early qualitative to quantitative studies of specific linguistic structures. However, the focus of these studies is usually on one aspect of an interlanguage instead of the whole system. The ideal object of interlanguage research is a second language (L2) learner language system, for only in this way can the entire L2 learning process can be examined. As a self-organizing and self-regulated system, the panorama of interlanguage can be revealed objectively through a complex network approach. In this study, we construct eight interlanguage dependency syntactic networks of varying proficiency levels and modalities, and conduct a quantitative study of respective network parameters. We find that all syntactic networks of Chinese L2 learners (English native speakers) initially present scale-free and small-world properties. Additionally, there is no sudden syntactic emergence in interlanguage with different modalities. This suggests varying regularities in the development of a syntactic network between interlanguage and native language acquisition. Moreover, the first language plays an important role in L2 development. The network parameters (<*k*>), *L, C, ND*, and *NC* can differentiate interlanguage modalities, and five quantitative parameters, <*k*>, *C, ND*, γ′, and *NC*, can indicate L2 proficiency.

## Introduction

The emergence of language faculty is of great significance in the process of human evolution (Deacon, 1997). It has been shown that the language development of children can be divided into babbling, lexical spurts, the two-word stage, and syntactic spurts. With limited time and language input, children can acquire pronunciation rules, amass large vocabularies, and master complex grammatical rules (Mackey, 1967; Radford, 1990). Some researchers contend that the innate language acquisition device (LAD) of the brain is integral to this process (Chomsky, 1966). LAD is based on natural universal grammar. Universal grammar is a natural system of principles, conditions, and rules shared by all human languages. The latter task of language acquisition is to assign values to the parameters of universal grammar through a language input (Particular Grammar, PG) (Chomsky, 1965).

In order to explicate the human language acquisition mechanism, researchers have explored the inherent processes of native language acquisition of children. The natural order of first language (L1) acquisition is consistent not only throughout the learning process but also in the acquisition of grammatical categories. Brown (1973) and De Villiers and De Villiers (1973) found that English children acquire morphemes in roughly the same order of grammatical items. In addition, negative sentences, interrogative sentences, double-object construction, and relative clauses are also acquired in roughly the same order (Klima and Bellugi, 1966; Cazden, 1972; Sheldon, 1974; Snyder and Stomswold, 1997; Campbell and Tomasello, 2001). Children acquire L1 grammatical rules in the same order as well, meaning there is likely a natural universal order in the L1 acquisition process. This provides empirical support for the innate nature of language competence, and the L1 acquisition process as likely constrained by UG (Universal Grammar) (Chomsky, 1965).

Interlanguage (hereafter IL) is a concept in the field of the second language (L2) acquisition. It refers to a natural language system produced by L2 learners when they are acquiring a new language (Richards et al., 1996). The mechanism of IL acquisition is an important topic in research on second language acquisition. Several studies on acquisition order in L1 since the 1970s have explored IL order acquisition. Numerous empirical studies show that although L2 learners differ in native language backgrounds and ages, their IL has a tendency to follow an order in the acquisition of specific syntactic structures, such as morphemes, negative sentences, and interrogations (Dulay and Burt, 1973; Bailey et al., 1974; Cazden et al., 1975; Wode, 1984; Goldschneider and DeKeyser, 2005). These studies have convinced some scholars that UG still dominates the second language acquisition process (Flynn, 1987; Thomas, 1991). Considering the fluctuations in the L2 learning process, researchers with varying views believe that L2 learners are affected by many initial conditions, such as L1, language usage experience, and psychological conditions. Moreover, they view L2 learning as a dynamic process (Larsen-Freeman, 1997; Ellis, 1998; De Bot et al., 2007; Larsen-Freeman and Cameron, 2008). L1 knowledge is an important source for L2 acquisition, as the role of universal grammar in second language acquisition may vary from that of native language acquisition of children (Bley-Vroman, 1989; Cook, 1991).

Discussions on the source of language acquisition have focused on morphemes or specific linguistic structures. Limited by the traditional research paradigm, few studies have examined the complete language system from the perspective of a syntactic network, and the source of the second language remains debated (Ellis, 2015).

In modern linguistics, Saussure (1959) has proposed that language is a system in which each linguistic unit is defined by its relations with other units. The idea that language is a network is accepted by most linguists. For example, proponents of Stratificational Grammar, Cognitive Grammar, and Construction Grammar regard language as a system (or network) that can be described by nodes and their relations (Lamb, 1966; Langacker, 1987; Goldberg, 1995; Larsen-Freeman and Cameron, 2008). It has been acknowledged that language is a complex (adaptive) system. A complex network cannot predict an entire behavior from its components. This is consistent with the view that “the whole is greater than the sum of the parts” in cognitive linguistics. Human language is also a typical complex system that shows a high degree of complexity at lexical, syntactic, and semantic levels. This means that it is difficult to adopt traditional linguistic research methods to study the overall characteristics of a language. Thus, complex networks are needed to study language (Liu, 2010).

It has been found that complex systems with different topologies in various fields, such as the World Wide Web, the biological food web, and social networks, show similar statistical patterns: the distances between the nodes in these networks hover around a tiny number, exhibiting a small-world effect. Meanwhile, the connectivity of the nodes in the network presents a power-law distribution (Martinez, 1992; Albert et al., 1999; Bearman et al., 2004). The application of the complex network approach in the field of linguistics has facilitated the quantitative study of the panorama of language. By analyzing complex networks, researchers have found that the syntactic networks of different language types have the same small-world and scale-free properties as other complex networks. Consequently, small-world and scale-free properties are seen as universal features of human language on a macro scale (Ferrer i Cancho et al., 2004; Cong and Liu, 2014). The small-world effect in language complex networks can be understood as the high efficiency of communication between nodes. Provided the language complex network is regarded as the network model for language knowledge, the high efficiency means that language knowledge is organized and is easily processed and retrieved (Liu, 2017). The scale-free property means that in a network, a minority of nodes have an extremely high degree, whereas a majority of nodes have a low degree. The power-law distribution, i.e., the Zipfian-like distribution, suggests “the principle of least effort” (Zipf, 1949). This is a balance between the demands of speaker/writer and hearer/reader to minimize the effort in language production and comprehension (Fan and Jiang, 2020). Parameters vary across complex networks, which means that properties of complex networks can be used to investigate the universality and individuality of complex networks (Albert and Barabási, 2002; Ferrer i Cancho, 2005; Liu, 2008; Liu and Xu, 2011; Cong and Liu, 2014; Amancio, 2015). Consequently, we can analyze the properties of syntactic networks of learners at different learning stages in order to identify developmental features of L2 learning. Corominas-Murtra et al. (2009) were the first to take the approach of a complex network to investigate the source of L1 acquisition. By studying development and changes in network parameters, they found that children can only produce scattered phrase structures up to around 24 months, and that their syntactic networks are pre-syntactic and tree-like. From around 2 years of age onward, children can output complete and syntactic sentences. This suggests that the syntactic development of L1 acquisition of children progresses from pre-syntactic organization to a scale-free and small-world complex syntactic network by leaps and bounds. It is believed that the language production of children experiences a qualitative leap from scattered phrase structures to complete and syntactical sentences under the condition of the limited given input. Barceló-Coblijn et al. (2012) and Barceló-Coblijn et al. (2019) found that regardless of which native language infants have acquired, their syntactic networks change in a similar way, from tree-like networks to small-world networks at a similar period (between 700 and 800 days). The nature of this transition offers support for the presence of an innate component (or a language-specific innate predisposition) that pervades the emergence of full syntax, and challenges usage-based theories of language acquisition (Corominas-Murtra et al., 2009, 2010; Barceló-Coblijn et al., 2012). In the field of L1 acquisition, the linguistic structures produced by children display a sharp syntactic transition at around 24 months, from chaotic word clusters to organized sentences. For example, prior to the transition, semantically degenerated elements (such as *it*) act as hubs that change from semantically degenerate to functional items (i.e., *a* or *the*) after the transition (Corominas-Murtra et al., 2009). Meanwhile, after the transition, the network also changes from a pre-syntactic tree-like network to a scale-free and small-word syntactic network. Moreover, the properties of “small world” and “scale-free” are universal features in adult human language syntactic networks. This indicates that syntactic abilities of children are beginning to approach those of adults. Therefore, the properties of “small world” and “scale-free” in syntactic networks can serve as metrics to measure the emergence of a syntactic network (Jiang et al., 2019).

For L2 complex systems, existing studies have focused on specific semantic and syntactic structures. For example, Mellow (2006, 2008) found that native Spanish speakers produced an expanding range of English constructions that also gradually grew in complexity. Williams and Kuribara (2008) studied the learning process of Japanese word order by native English speakers. Ellis and Larsen-Freeman (2009) explored the acquisition of English verb-argument structures by English bilinguals. Verspoor et al. (2012) investigated the development of sentence, phrase, and word level in English writing produced by Dutch children. Borodkin et al. (2016) probed into the organization of mental lexicons of Hebrew L2 speakers. These studies suggest that L2 acquisition is likely to rely on the language experience and interaction by means of outside language input, and that L2 language development is based on language experience and usage indicates the existence of a regular pattern of dynamical system theory (Ellis and Larsen-Freeman, 2009; Ellis, 2012). Based on the complex network approach, a recent study has examined the overall syntactic development of written English interlanguage. It found that unlike the L1 learning of native English children, English L2 learning is characterized by a gradual approximation to the target language instead of a sudden emergence at a point (Jiang et al., 2019). However, this study on the syntactic development of the whole interlanguage system has several deficiencies.

Jiang et al. (2019) examined the writing corpus of English learners, and Corominas-Murtra et al. (2009) studied the oral production of English native children. Previous studies have found differences in the syntactic performance across modalities and genres in both first language systems and interlanguage systems (Biber, 1988; Kormos, 2014; Biber et al., 2016; Qin and Uccelli, 2016; Zalbidea, 2017; Bulté and Roothooft, 2020). Cognitive processes and production patterns are inconsistent across modalities; spoken language is a reflection of the process of language construction, whereas written language is a revised and polished product (Halliday, 1989; Levelt, 1989; Kellogg, 2001; Cutting, 2011). The time pressure and cognitive burden of outputting oral language (on-line processing) is heavier than that of writing (off-line processing) (Grabowski, 2007). From the perspective of complex networks, Liu (2008), and Chen and Liu (2013) also found variation in several important network parameters across modalities and genres. Can the conclusion drawn by analyzing the corpus of L2 writing in Jiang et al. (2019) be compared with the conclusion drawn by the analysis of the oral language of children produced in Corominas-Murtra et al. (2009)? This needs further discussion. Do the conclusions of studies of written interlanguage apply to oral interlanguage? Are there differences in syntactic networks between written and oral interlanguage? If there are differences between the two modalities, which syntactic network parameters can differentiate the modality features of the L2? These issues remain unsolved in the research on the similarities and differences of interlanguage features across modalities from the perspective of a syntactic network.

Jiang et al. (2019) focused on the development of a syntactic network of inflectional English as an interlanguage. Meanwhile, the interlanguage of other typological languages has received scant attention. The question of whether or not the previous findings are coincidental requires further verification of interlanguage in other languages, such as the isolated Chinese language. What are the developmental features of the syntactic network of Chinese interlanguage? It is necessary to expand the scope of the research sample to explore whether there is a phenomenon of emergence in the syntactic network of interlanguage.

Based on the dynamic development of interlanguage (Larsen-Freeman, 1997), one of the most important topics in the field of L2 acquisition is finding metrics for measuring the development of second language proficiency. Jiang et al. (2019) focused on the small-world and scale-free properties in English interlanguage, but they did not directly relate the development of syntactic network parameters to the language proficiency of learners. In their study, no further statistical tests were conducted to determine which network parameters can predict interlanguage proficiency. From the perspective of syntactic networks, what parameters can be used to measure the syntactic development of interlanguage? Can the metrics be used to measure the syntactic development of different modalities in the same vein? Solving these problems necessitates studying the interlanguage of an L2 learner along with comparable language modalities.

To fill the above-mentioned gaps, in this study, we have selected written and oral corpora of English native speaking Chinese second language learners (ECSL learners) to investigate the regularity of the syntactic network of the interlanguage system. We first discuss the origin of the interlanguage acquisition mechanism, and then the relationship between complex network statistical parameters and interlanguage modalities and language proficiency. The research questions are as follows:

(1) From the perspective of the overall interlanguage syntactic network development, do complex networks of Chinese interlanguage of different modalities emerge suddenly? Are syntactic developmental pattern and mechanism source of L2 acquisition similar to those of L1 acquisition?
(2) Are there any differences between the interlanguage complex network properties across modalities? If there are differences, what complex network properties can differentiate interlanguage modalities?
(3) What properties of the syntactic network can be used to measure the level of interlanguage development in different modalities? Are there differences in the network parameters used to measure interlanguage proficiency across modalities?

## Materials and Methods

### Participants and Materials

The writing (W) corpora used in this study are those of ECSL learners in-class compositions. A total of 190 authors participated in this study, aged between 20 and 25. Each was majoring in the Chinese language at a university in Beijing. All of them had begun learning Chinese when they were admitted to college. The compositions of native language backgrounds of Chinese learners were classified into four proficiency levels (P1–P4). Every composition at each level was written by its mid-level students. According to these levels, the written corpus was divided into four sub-corpora, i.e., W1, W2, W3, and W4, with each level containing 5,000 words after removing the punctuation. The oral (O) interlanguage corpus comes from the topic-based speaking tests of ECSL learners. It was also divided into four sub-corpora, i.e., O1, O2, O3, and O4, each having 5,000 words. The topic of each text was related to the daily lives of CSL learners. All composites were narratives with topics familiar to the L2 learners, such as “my daily life,” “my hobbies,” and “a person I am familiar with.” After completing the written and oral tasks, they allowed researchers to collect their writing and oral corpora to study. In order to investigate the learnability issues in L2 acquisition, we selected Chinese (target language, hereafter TL) written and oral corpora as the L2 learning research reference. We randomly selected the corpus of contemporary classical Chinese novels based on narration as the written corpus of Chinese native speakers (WN), and took the transcriptions of *Shi-hua-shi-shuo* (*straight talk*), a famous Chinese talk show, as the oral corpus (ON) of Chinese native speakers. These two contrastive TL corpora were used as a reference with each corpus consisting of around 5,000 tokens. The number of texts, years of Chinese learning, HSK (Chinese Proficiency Test, HSK, from level I to VI, an international standardized test that assesses aptitudes of non-native Chinese speakers in using the Chinese language in their daily, academic, and professional lives), level, and L2 vocabulary for each grade are shown in Table 1.

**Table 1:** Basic data for each corpus.

**Modalities**	**L2 proficiency**	**Participants**	**Years of Chinese learning**	**HSK level**	**Vocabulary size**
Writing	W1	46	0.5–1	II	500
	W2	24	1.5–2	III	800
	W3	14	2.5–3	IV	2,000
	W4	8	3.5–4	V–VI	4,000
Oral	O1	34	0.5–1	II	500
	O2	24	1.5–2	III	800
	O3	17	2.5–3	IV	2,000
	O4	12	3.5–4	V–VI	4,000

### Building Syntactic Dependency Networks

Like a natural linguistic network, the Chinese interlanguage complex network consists of vertices (nodes) and edges (Newman, 2010). In linguistic complex networks, nodes represent language units, and edges represent syntactic relationships between language units (Liu, 2008). Previous studies have shown that dependency analysis is an effective way to construct a syntactic network for analyzing the development of syntactic structures among language learners (Corominas-Murtra et al., 2009; Jiang et al., 2019). The syntactic dependency network is a dynamic language network based on words and their syntactic dependency relationships in real contexts, transferred from a syntactic dependency treebank. Treebanks tag sentences word-for-word in the frame of syntactic dependency, which can determine the syntactic dependency between words in sentences. Dependency analysis is based on a binary grammatical relationship between words, so it is convenient to transform the dependency analysis of a sentence into a network representation (Hudson, 2007). In addition, dependency grammar has been shown to be more suitable for researching language acquisition for learner language involving syntactic mistakes (Jiang and Ouyang, 2017).

**Figure 1:**
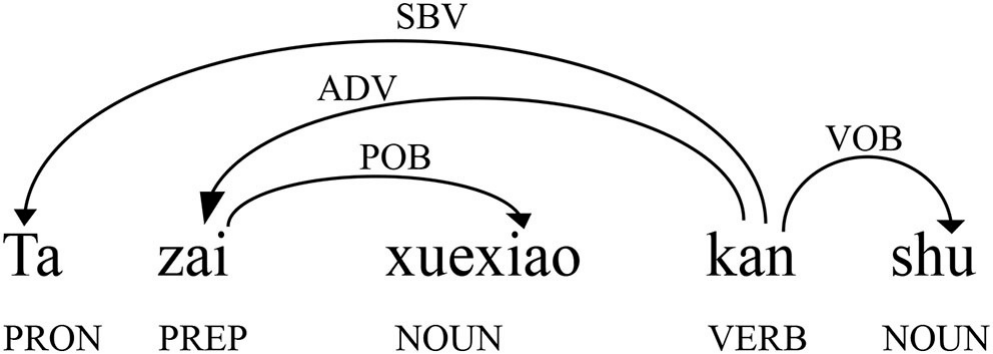
Dependency structure of the sentence *Ta zai xuexiao kan shu* (“he reads books at school”). The word pointed by the arrow is the dependent.

Dependency grammar holds that words in a sentence are connected by syntactic dependency relations (Hudson, 2007). The dependency relation is formed between two asymmetrical, directional syntactically related structural elements. One is the governor (the head word), and the other is the dependent (the word governed by, or dependent on, the head word). The task of syntactic analysis is to determine the dependency relationship between the governor and the dependent (Liu, 2009a). According to these properties of dependency grammar, we can construct dependency structures marked by directed arcs. Figure 1 shows the dependency analysis of the sentence *Ta Zai Xuexiao Kan Shu* (“He reads books at school”). The syntax labels above the arc indicating the dependency relation between the two words are shown by the arrow pointing from the governor to the dependent. From a macroscopic syntactic complex network perspective, the governor and the dependent are the vertices in networks, and their dependency relations above the directed arcs are the edges in the syntactic network.

The basic dependency-annotated process can be divided into word segmentation, part-of-speech (POS) tagging, dependency syntactic tagging, and error tagging. First, the part of speech annotation and the dependency relation tagging were automatically completed by Language Technology Platform (LTP) (Che et al., 2010), a Chinese processing platform developed by the Research Center for Social Computing and Information Retrieval at Harbin Institute of Technology. Then, manual proofreading and error labeling were conducted on the basis of preliminary machine labeling. We retained the dependency relations of syntactic errors of L2 learners to ensure not only the efficiency and accuracy of the annotation to reflect the real proficiency of CSL learners but also the reliability research results of this study. We then used Python scripts to convert the information in Figure 1 into Table 2. As shown in Table 2, each row has a dependency pair consisting of three main parts: the dependent, the governor, and the dependency type. If a sentence contains *n* words, it has *n*−1 dependencies. From the perspective of a syntactic network, it can be transformed into a graph with *n* nodes and *n*−1 edges.

**Table 2:** Annotation of the sample sentences in Chinese.

**Sentence**	**Dependent**			**Governor**			**Dependency type**
	**Order number**	**Word**	**POS**	**Order number**	**Word**	**POS**	
s1	1	Ta/He	r	2	Kan/reads	v	SBV
s1	2	Kan/reads	v	0	/	/	HED
s1	3	Shu/books	n	2	Kan/reads	v	VOB
s2	1	Ta/He	r	4	Kan/reads	v	SBV
s2	2	Zai/at	p	4	Kan/reads	v	ADV
s2	3	Xuexiao/school	n	2	Zai/at	p	POB
s2	4	Kan/reads	v	0	/	/	HED
s2	5	Shu/books	n	4	Kan/reads	v	VOB

**Figure 2:**
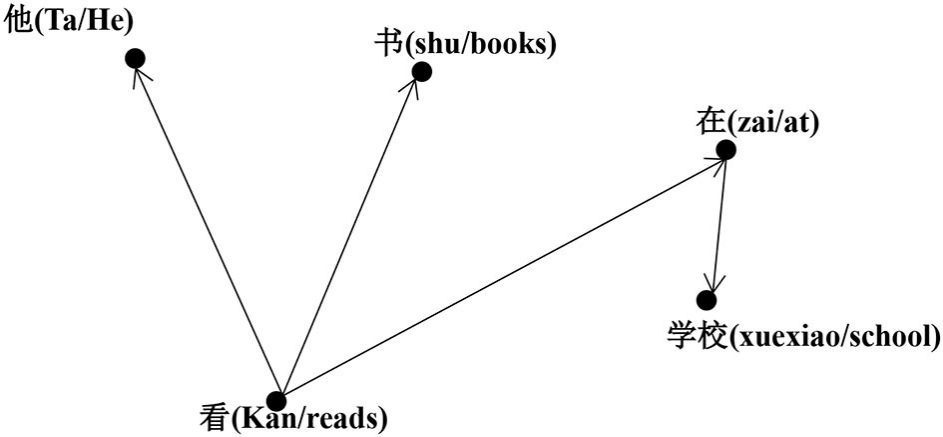
The syntactic dependency network of two sentences *Ta kan shu* (“he reads books”) and *Ta zai xuexiao kan shu* (“he reads books at school”).

The dependency analysis set consisting of the two sentences in Table 2 has been represented by Create Pajek^1^ as the syntactic directed network shown in Figure 2. In this figure, the arrows from the governor to the dependent indicate the syntactic relationship. The network analysis calculation in this study was done according to the undirected network. We used Pajek (Nooy et al., 2011) to calculate the parameters of a total of 10 syntactic networks in eight interlanguages and two in native Chinese.

### Target Network Properties to Analyze

After constructing the language complex network model, we can use appropriate complex network properties to analyze features of interlanguage networks. This can reflect the overall characteristics of the corresponding language subsystem. For evaluating the complexity of a network, the most investigated network indicators are its average path length (*L*), clustering coefficient (*C*), average degree (<*k*>), and degree distribution [P(*k*)]. We can generalize the nature of a network in terms of whether it is a small-world or a scale-free network (Albert and Barabási, 2002).

#### Network Indicators Related to Degree (*k*)

In a network, non-isolated vertices are connected to one or several other vertices. The node degree *i* (*k*_*i*_) is the total number of edges that the vertex has, which is the number of other vertices that directly connect to it. The degree of *Kan* (“read”), as shown in Figure 2, is three. The average degree <*k*> of a network is the mean degree of its vertices, which represents the estimated value of the syntactic valence of the words corresponding to any vertex. As shown in Figure 2, for example, the syntactic network has five vertices, i.e., *Ta, Zai, Xuexiao, Kan*, and *Shu*, and their degrees are 1, 1, 1, 3, and 1, respectively. Accordingly, the total number of degrees for these syntactic networks is 7, and the average degree of the syntactic network is 1.4 (7/5). The degree distribution [P(*k*)] is the probability distribution of the degree of vertices in a network. The degree distribution of a random network follows the Poisson distribution, while the degree distribution in real networks generally fits the power-law distribution. A network that obeys a power-law distribution is called a scale-free network, following the formula *P(k)*~*k*^−*y*^. The degree distribution fits the power law for some constant exponents γ, and then its corresponding cumulative degree distribution for reducing the noise in the tail and making exponents of the power law more precise follows Zipf's law, with exponent γ′ equaling γ−1 (Jiang et al., 2019).

#### Average Path Length (*L*) and Clustering Coefficient (*C*)

The distance *d*_*ij*_ between two vertices *i* and *j* in a network is the number of edges on the shortest path connecting the two vertices. For example, as shown in Figure 2, the shortest paths between *Ta* and *Zai*, and *Shu* and *Xuexiao* are 2 and 3, respectively. Therefore, the average path length *L* of the network is the average distance between any two vertices:

$$\begin{array}{l} L=\frac{1}{\frac{1}{2}N\;\left(N-1\right)}\underset{i>j}{\sum }dij \end{array}$$

In the above formula, *N* is the number of vertices in the network, and *d*_*ij*_ is the distance between vertex *i* and vertex *j*, which can be represented by the number of edges in the shortest path between two vertices.

For a given vertex *i*, there may also be edges between its adjacent nodes (other vertices directly connected to it, of which there are *k*_*i*_ vertices). In other words, its adjacent vertices may be adjacent to each other. The complex network shown in Figure 2 does not have three vertices connected to each other, so its clustering coefficient is 0. The probability of there being edges between any pair of adjacent vertices of vertex *i* is its clustering coefficient (*C*_*i*_) (Newman, 2010). If a vertex *i* has *ki* edges connected to other vertices, then this vertex and these vertices form a sub-network (or clustering). If *Ei* is considered to be the actual number of edges between the *ki* vertices, then the ratio of *Ei* to the maximum number of edges available between the *ki* vertices, *ki* (*ki*−1)/2 is the clustering coefficient *Ci* of vertex *i*:

$$\begin{array}{l} C_{i}=\frac{2Ei}{ki\left(ki-1\right)} \end{array}$$

The clustering coefficient *C* of the whole network is the average of the clustering coefficient *Ci* of all vertices, as represented by

$$\begin{array}{l} C=\frac{1}{N}\overset{N}{\underset{i=1}{\sum }}Ci \end{array}$$

The average path length and clustering coefficient of the network reveal whether the network has the small-world property. Complex networks with the small-world property have both a small average path length and far greater clustering coefficients compared with those of random networks (Watts and Strogatz, 1998; Cong and Liu, 2014).

#### Network Density (ND) and Network Centralization (NC)

Network density (ND) reflects the probability of there being edges between any pair of vertices. This represents the degree of compactness for each vertex in a graph. The ratio of the actual number of edges (*M*) to the theoretical maximum of the number of edges ($C_{N}^{2}$) in the network is the ND:

$$\begin{array}{l} ND=\frac{M}{C_{N}^{2}} \end{array}$$

where

$$\begin{array}{l} C_{N}^{2}=\frac{N\left(N-1\right)}{2} \end{array}$$

Network degree centralization (NC) represents the centralization of the network (Dong and Horvath, 2007). The central potential of degrees reflects the relative intensity of the central vertex in a network. It is another important parameter to consider. For Chinese syntactic networks, function words are likely to be the central nodes of a network (Chen and Liu, 2016). The calculation formula is (Liu, 2017).

$$\begin{array}{l} NC=\frac{N}{N-1}\left(\frac{k_{max}}{N-1}-\rho \right)\approx \frac{k_{max}}{N}-\rho \end{array}$$

where *k*_*max*_ is the maximum vertex degree of the network, and ρ is the network density calculated above.

In conclusion, the degrees of vertices in a scale-free network generally follow a power-law distribution, i.e., a Zipfian-like distribution, which suggests that it follows “the principle of least effort” (Zipf, 1949). This is a balance between the demand of speaker/writer and hearer/reader to minimize the effort in language production and comprehension (Fan and Jiang, 2020). The value of average path length and clustering coefficient can judge whether the network has the small-world property. The small-world effect in language complex networks can be understood as the high efficiency of communication between nodes. Provided that a language complex network is regarded as the network model for language knowledge, the high efficiency means that language knowledge is organized, and easily processed and retrieved (Liu, 2017). The higher the network density (ρ) value, the denser the edges of the network, and vice versa. Complex language networks are generally sparse, which means that the probability of a certain relation (i.e., a syntactic dependency relation) between any two language units in a language subsystem is miniscule (Liu, 2017). The central potential of degrees (NC) contributes to finding the central node in the network. For a dynamic language network, the central potential of the degree reflects the strength of the combining ability of the central node. The linguistic unit binding strength corresponding to the central node is high whenever the NC value is high.

## Results

### General Information on Syntactic Networks of CSL Learners

Table 3 presents basic information on syntactic networks of CSL learners and Chinese native speakers, such as vertices representing how many word types and word tokens there are, i.e., the total word count. As shown, the vertices of Chinese interlanguage across modalities gradually increase as language proficiency improves. Vertices in the syntactic network are word types. The number of vertices in the syntactic network gradually increases with improvement in Chinese proficiency. This means that English native speakers produce more word types as their proficiency advances in both written and oral languages, but the language proficiency of CSL learners at P4 is still lower than that of Chinese native speakers. For example, CSL learners had 773 vertices at P4 in oral production, but native speakers had 1,037. In addition, vertices in written Chinese interlanguage are higher than those of the oral throughout the learning process. The word token count in written Chinese interlanguage gyrates upward toward 5,224 from P1 to P4. Meanwhile, the total word count for oral interlanguage showed an increasing trend.

**Table 3:** Basic information for each network.

**Network**	**W1**	**W2**	**W3**	**W4**	**WN**	**O1**	**O2**	**O3**	**O4**	**ON**
Vertices	1,001	1,168	1,227	1,367	1,710	616	672	733	773	1,037
Word tokens	5,167	5,154	5,085	5,224	5,164	4,910	4,986	5,142	5,319	5,094

**Figure 3:**
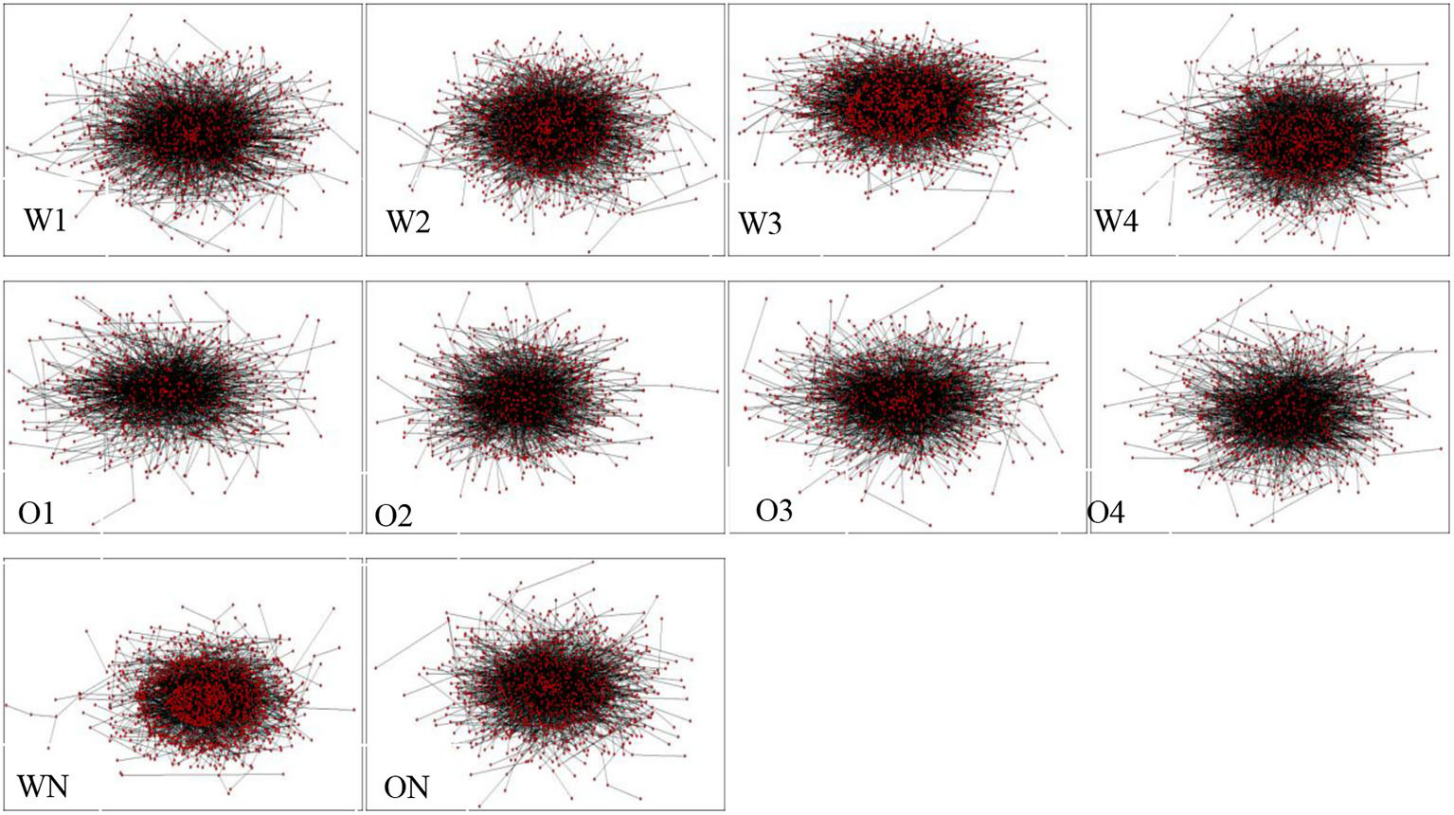
CSL learners and native speakers' syntactic dependency networks.

Figure 3 shows the syntactic network of ECSL learners across modalities and language proficiencies. The edges between vertices represent word types and dependency syntactic relations, respectively. Correlation analysis shows that the number of vertices and edges and Chinese proficiency of the two modalities are highly correlated with high *R*^2^ values (*ps* < 0.05), indicating that the vertices and edges of the Chinese interlanguage syntactic network with different modalities become denser with improvement in Chinese proficiency.

### The Properties of Syntactic Networks of CSL Learners

Scale-freeness and small-worldness are important properties in the complex network. We can derive parameter values for interlanguage syntax network-related properties with the help of Pajek, as shown in Table 4.

**Table 4:** Major parameters of the 10 networks.

**Networks**	*****γ^,^*****	***R*^**2**^**	***<k>***	***C***	***L***	***ND***	***NC***
W1	1.057	0.9659	6.11	0.219	3.019	0.009	0.19
W2	1.132	0.9713	5.914	0.211	3.008	0.007	0.197
W3	1.329	0.9804	5.514	0.211	3.062	0.006	0.231
W4	1.33	0.9761	5.482	0.162	3.082	0.005	0.246
WN	1.481	0.9727	4.815	0.131	3.651	0.003	0.291
O1	1.243	0.9711	6.979	0.333	3.298	0.02	0.144
O2	1.272	0.9643	6.906	0.29	3.331	0.018	0.152
O3	1.272	0.9711	6.821	0.28	3.290	0.016	0.169
O4	1.282	0.9613	6.617	0.264	3.174	0.015	0.171
ON	1.307	0.9808	6.002	0.204	3.279	0.008	0.198

**Figure 4:**
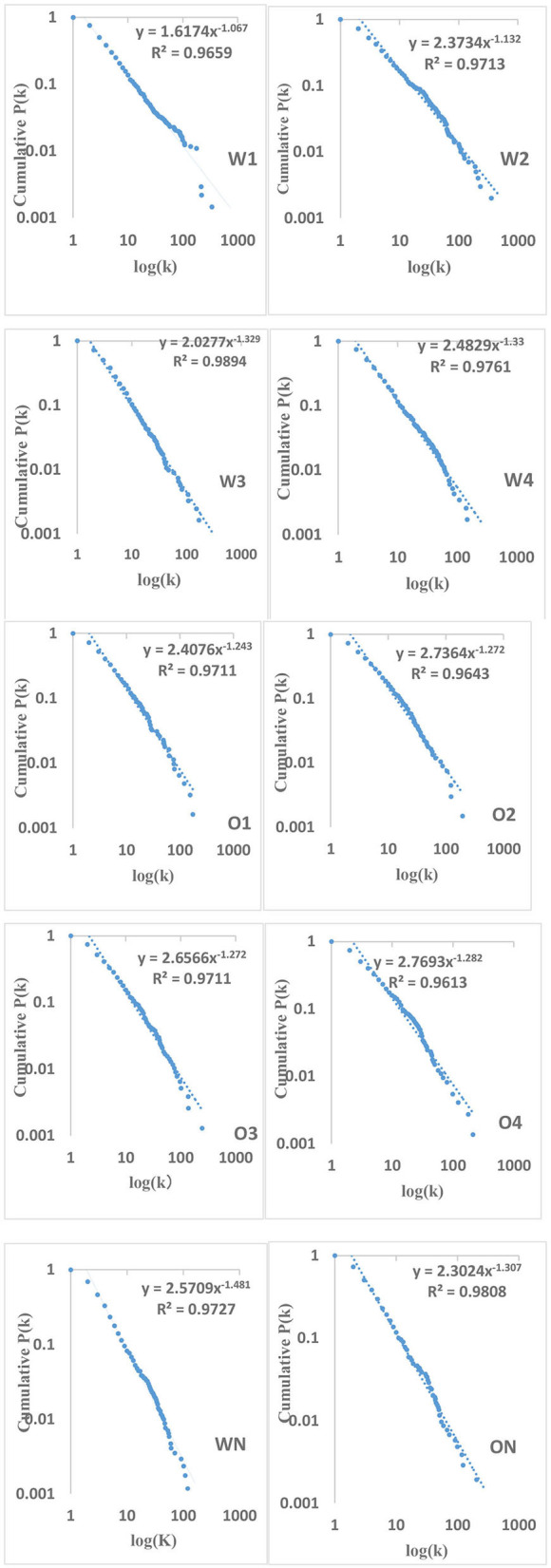
Syntactic dependency networks' cumulative degree distributions.

The distribution of degrees of vertices in a complex network and a random network generally follows the power-law distribution and Poisson distribution, respectively (Barabási and Albert, 1999; Newman, 2003, 2010). A network with the power-law distribution of degrees has the property of scale-freeness, showing that only few linguistic units have the strong general syntactic capacity to combine with other linguistic units (words), while most other linguistic units have weaker combining ability (Barabási and Albert, 1999). To examine whether there is scale-freeness in the syntactic networks of CSL learners, it is necessary to examine the regularity of degree distribution (<*k*>) in syntactic networks of CSL learners with varying language proficiencies. Therefore, the regularities of degree distributions in networks of eight CSL learners with varying proficiencies were first extracted, and then we compared these with those of corresponding random networks. The cumulative degree distributions were chosen to reduce the noise in the long tail, and to increase the precision of the exponents of the power-law. Figure 4 shows the results of 10 cumulative degree distributions, the determination of which is each well-fitted by a power law with determination coefficients *R*^2^ above 0.9. This indicates that Chinese interlanguage syntactic networks representing different language proficiencies each fit the power-law distribution, displaying Zipfian-like distributions. Meanwhile, the 10 sets of data of L2 learners and native speakers with two different modalities fit the Poisson unsuccessfully (the determination coefficients *R*^2^ of W1, W2, W3, W4, and WN are 0.1361, −0.0056, 0.5099, 0.3852, and −41.5262, and those of O1, O2, O3, O4, and ON are 0.3591, 0.2772, 0.2847, 0.2724, and 0.4623, respectively). Additionally, using multiple open source packages of R statistical programming software ver-3.6.3., we fit the 10 sets of data with the Poisson distribution model and the power-law distribution model, so as to observe their Akaike Information Criterion (AIC) value. The results show that the AIC value of the Poisson distribution model of 10 sets of data are all Inf, which means that the AIC values are infinite, and all analyses statistical significance of the Poisson distribution model are accepted as *ps* > 0.05. However, the AIC values of the power-law distribution model of the 10 sets of data are all negative (AIC values of W1, W2, W3, W4, and WN are −315.363, −390.6772, −300.4212, −338.9469, and −62.9342 and those of O1, O2, O3, O4, and ON are −247.8501, −268.8115, −298.5522, −270.203, and −289.8271, respectively), and all analyses statistical significance of the power-law distribution model are accepted as *ps* < 0.05. In short, AIC values of the power law distribution model are much smaller than those of the Poisson distribution model, which indicates the degree distributions of the vertices in 10 of networks we built follow the power-law distribution instead of the Poisson distribution. This suggests that all CSL learners' syntactic networks exhibit the scale-free property. In addition, <*k*> values for the two modalities produced by CSL learners with different proficiencies are negatively correlated with their Chinese proficiency. The regression equation for <*k*> in the written corpus is *y* = 6.474–0.302^*^ *x, p* = 0.01, *R*^2^ = 0.922, and that in the oral corpus is *y* = 7.3379–0.2243^*^x, p = 0.038, *R*^2^ = 0.8077. Additionally, a *t*-test shows that the <*k*> value in writing production differs from that of spoken production (*t* = 6.234, *df* = 4.454, *p* < 0.005). As shown in Table 4, the power law distribution index γ′ of the degree in interlanguage with different modalities increases with their increasing second language proficiency, but it does not reach the target language level. The regression equation for written production of CSL learners is *y* = 0.952 +0.105^*^x, p = 0.006, *R*^2^ = 0.944, and that of spoken production is *y* = 1.234 +0.014^*^x, p = 0.014, *R*^2^ = 0.901, whereas there is no significant difference between interlanguage across modalities (*p* = 0.46).

A small-world network has shorter average path length (*L*) and a higher clustering coefficient (*C*) than its random network (Watts and Strogatz, 1998). As shown in Table 4, the average path lengths extracted from networks of eight CSL learners are between 3.008 and 3.331, and the value of *L* in the corresponding eight random networks is in the range of 3.703–5.79, higher than that of CSL learners at all levels. There is no significant difference between writing production (*p* = 0.125) and spoken production (*p* = 0.37) of CSL learners in terms of the value of *L*. The *L* of oral interlanguage (3.273) is 0.23 higher than that of written interlanguage (3.043) (*t* = 5.991, *df* = 6, *p* < 0.005), but the difference is lower than that of native Chinese oral and written productions (0.372). In addition, the values of *L* for interlanguage across modalities are also less than those of Chinese native speakers. Secondly, for the parameter *C*, the value ranges in written and spoken Chinese interlanguage are 0.162–0.333, while those of the eight corresponding random networks are only 0.002–0.009, far lower than those of CSL. The values of *C* for written and oral interlanguage have a significant negative correlation with Chinese proficiency. The result of regression equations of the written interlanguage is *y* = 0.254 −0.023^*^x, p = 0.025, *R*^2^ = 0.853, and that of the oral interlanguage includes *y* = 0.359 −0.028^*^x, p = 0.01, *R*^2^ = 0.919. At the same time, the *C* of writing of CSL learners (0.201) is lower than that of oral production (0.292) (*t* = 4.619, *df* = 6, *p* < 0.005). At advanced levels, CSL learners do not reach the target language level in the same way. Compared with corresponding random networks, the results show that all syntactic networks of CSL learners have far greater clustering coefficients and smaller average path lengths. According to Watts and Strogatz (1998), the syntactic networks produced by CSL learners display the small-world property at the onset of Chinese learning.

### The Centrality of Syntactic Networks of CSL Learners

According to Formulas (4) and (5), we calculate the values of *ND* and *NC* for Chinese interlanguage across four levels and two modalities. The results are shown in Table 4. As shown, the *ND* and *NC* of the two interlanguage modalities have changed significantly with Chinese proficiency improvement. First, the *ND* for Chinese writing and oral interlanguage decreased with improved Chinese levels; the *ND* for written interlanguage is lower than that of spoken written interlanguage throughout the acquisition process, and neither reach the target language level at P4. Moreover, the regression equation for written production is *y* = 0.01 −0.0014^*^x, p = 0.001, *R*^2^ = 0.98, and that of oral interlanguage is *y* = 0.0235 −0.0027^*^x, p = 0.019, *R*^2^ = 0.876. In addition, there is a significant difference between writing and oral production for interlanguage (*t* = 7.503, *df* = 6, *p* < 0.001). Additionally, the overall *ND* for writing (0.0068) is less than that for speaking (0.0173), which is consistent with the results for Chinese native speakers. Second, the changing trends in *NC* and *ND* are opposite. The *NC*s of written and spoken interlanguage both increase with the improvement in Chinese proficiency, while the regression equation of the former is *y* = 0.156 +0.025^*^x, p = 0.005, *R*^2^ = 0.946, and it is *y* = 0.129 +0.013^*^x, *p* = 0.008, *R*^2^ = 0.93 for the latter. With regard to *NC*, the value for writing is significantly higher than that for speaking, which is displayed both in Chinese interlanguage and Chinese. Further statistical testing shows that written interlanguage is 0.057 higher than oral interlanguage (*t* = 3.814, *df* = 4.36, *p* < 0.05).

## Discussion

### The Properties of Syntax Networks of Interlanguage

According to Corominas-Murtra et al. (2009), although vocabularies of children are inferior to those of adults, a sharp transition in syntax, occurring around 24 months, is close to adult proficiency. From the perspective of the complex system, the syntactic development of the native language of children witnesses emergence, signaling the pivot from the pre-syntactic organization to a scale-free and small-world syntactic network. The authors suggest that this phenomenon cannot be explained merely by self-organizing or external driving mechanisms, such as communication constraints between individuals; on the contrary, innate mechanisms of language acquisition are likely functioning.

This study expands the sample of interlanguage types (Chinese) and investigates the syntactic network properties of an interlanguage with various modalities. The results indicate that there is no pivot and emergence in syntactic networks, across either written or oral Chinese interlanguage. Throughout the course of an acquisition, Chinese interlanguage networks all exhibit the properties of scale-freeness and small-worldness, i.e., the power-law distribution fitting coefficient *R*^2^ of degrees for the eight interlanguage syntactic networks is above 0.9. This differs from the degrees in corresponding random syntactic networks following a binomial distribution. Thus, it can be concluded that all the eight Chinese interlanguage syntactic networks have a scale-free property. Put differently, L2 learners with different L2 proficiencies follow the principle of least effort to construct their respective written and oral interlanguage systems. Furthermore, interlanguage networks have small *L* and far greater *C* than the random network, demonstrating that all the eight interlanguage systems feature small-worldness. This demonstrates that the language knowledge in interlanguage also has an efficient organization system like that of natural languages. This finding verifies and supplements Jiang et al. (2019), who investigated syntactic networks of English foreign language learners. Furthermore, we confirm that there is no sudden emergence in the syntactic development of writing, or in the oral corpus of interlanguage. This is applicable not only to the inflectional language of English as an interlanguage, but also to the isolating language of Chinese as an interlanguage. This suggests that there may be variation in language acquisition mechanisms between L2 and L1 learners. Moreover, the innate language acquisition device may play a disparate role in L2 acquisition. By and large, L1 acquisition and L2 acquisition are intrinsically different. All the corpora in this study were produced by adult L2 learners. Of note, L2 learners begin L2 learning with a parasitic lexicon, parasitic phonology, and parasitic set of grammatical constructs (MacWhinney, 1997). Thus, L1 conceptual knowledge indeed influences L2 acquisition (Türker, 2015).

Chinese is an isolating language that relies on function words and word order rather than on rich morphological information. This is the opposite of what English, an inflectional language, does (Li and Thompson, 1981). Although the two languages differ in terms of their surface grammatical devices, their deep semantic structures contain similarities, such as that semantic case indicates deep structures, and the internal semantic structures are language-universal (Fillmore, 1968). This is due to the basic human universal cognitive abilities (Palmer, 2006). Adjemian (1976) pointed out that interlanguage is peculiar in being permeable. This means learners can transfer grammatical properties from L1 into interlanguage. Existing L1 knowledge in the minds of L2 learners can facilitate their L2 acquisition to an extent, and the universal properties of language can also facilitate the integration of the native language with the target language. Therefore, neither English nor Chinese interlanguage shows sudden emergence of a syntactic system because the L1 knowledge of L2 learners provides much experience and reference for L2 processing. We contend that native language knowledge, rather than universal grammar, is an important mechanism device of L2 acquisition (Cook, 1991).

### Differences Between L2 Modalities of Syntactic Network Properties

Previous studies have shown that syntactic network parameters vary across modalities (Liu, 2008), and that the modality also has a significant effect on L2 performance (Cho, 2018). We also find that, like natural language, there are significant differences in the parameters of interlanguage syntactic networks. The results in Table 4 show significant differences between writing and oral interlanguage on <*k*>, *L, C, ND*, and *NC*.

**Figure 5:**
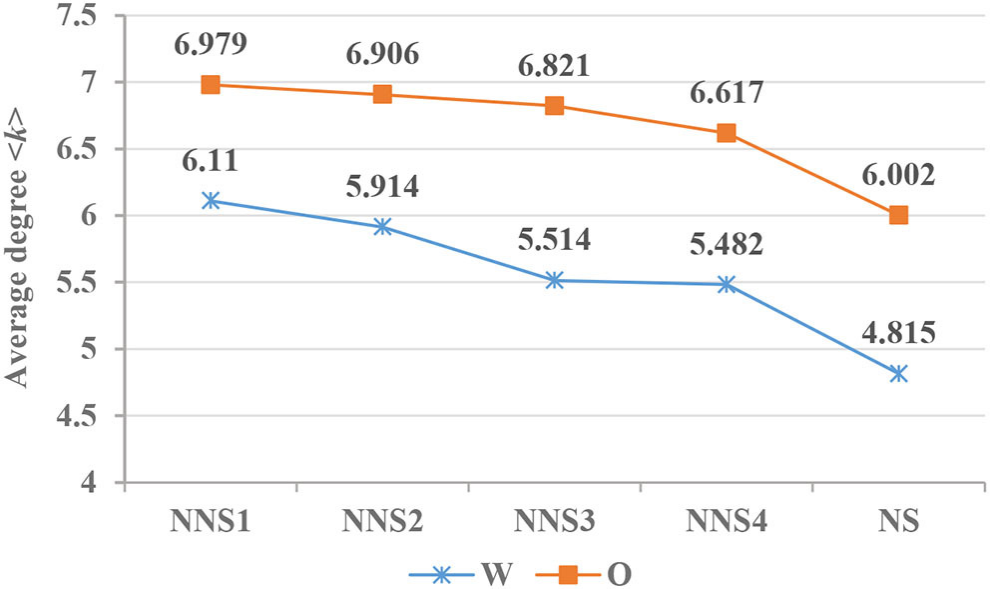
Changes in <*k*> in CSL learners' and native speakers' syntax networks. NNS1–NNS4, non-native speakers with low to high L2 language proficiency; NS, native speakers.

According to Figure 5, the value of spoken Chinese <*k*> (6.002) is higher than that of written Chinese <*k*> (4.815). Likewise, Liu (2008) has found that the value of <*k*> for spoken Chinese is higher than that for written Chinese. The <*k*> of the syntactic network not only reveals the lexical richness but also reflects the average combination ability of syntactic units (Liu, 2009b, 2017). First, on the condition of similar word token counts, the higher the mean degree, the lower the lexical richness of the corresponding corpus (Chen and Liu, 2013). The <*k*> (6.831) of oral interlanguage is not only higher than that of writing (5.755) on the average level but also for the entire learning process. This indicates that the written vocabularies of L2 learners are richer than their oral vocabularies, as has been claimed by Kormos and Trebits (2012) and Zalbidea (2017). Second, in terms of syntactic connectivity, highly connected words tend not to be interconnected (Ferrer i Cancho et al., 2004). In Chinese, function words, such as prepositions, conjunctions, and auxiliary and modal particles, are the most connected word types that do not form syntactic dependency among themselves and only act as intermediate words in a syntactic network (Chen and Liu, 2016). Chen and Liu (2016) found that the number of *de* (“of”) and *zai* (“in”) in writing production of CSL learners is higher than that in oral production. In addition, degrees of *de* are higher than those of *zai*, which is also found in native the production of Chinese speakers (Chen and Liu, 2016). For example, the total number of *de* in interlanguage's writing is 1.67 times that of in the oral corpus. Therefore, on average, each vertex in oral interlanguage has a syntactic relationship with 6–7 other vertices, while written language has more central nodes, except in the initial stage where the average vertex has a syntactic relationship with 5–6 other vertices.

**Figure 6:**
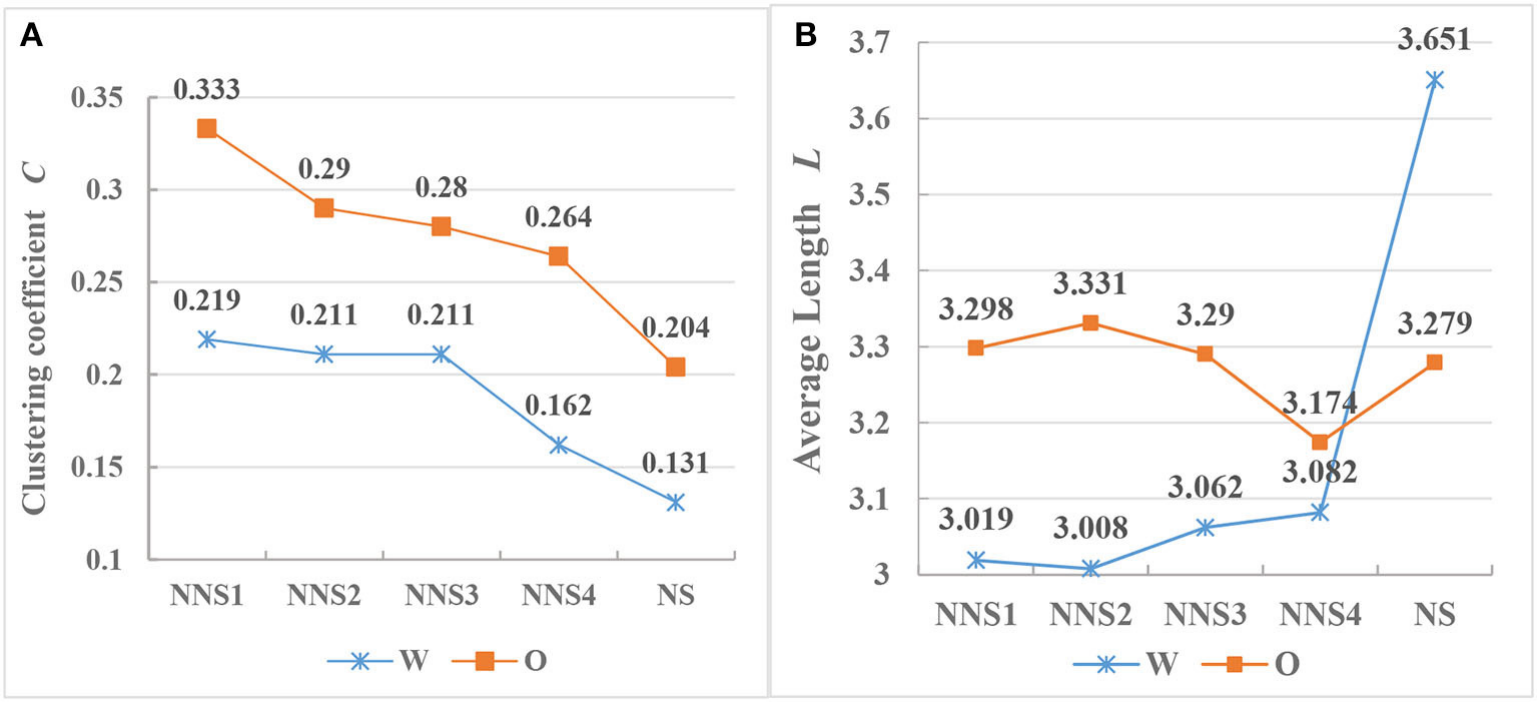
This is a figure with sub figures, **(A)** shows the values of *C* in CSL learners' and Chinese native speakers' syntax networks, **(B)** displays the values of *L* in CSL learners' and Chinese native speakers' syntax networks.

Liu (2008) showed that the *C* of written Chinese is less than that of oral Chinese, and that the *L* of the writing exceeds that of the oral. The Chinese network parameters can be viewed in the same light. With regard to the interlanguage, both written and oral networks have small-world properties. Meanwhile, there is a statistically significant difference between their *C* and *L* and those of natural language. The clustering coefficient is used to measure the tendency of aggregation or the tendency of forming clusters. It is defined as the probability of two neighboring vertices of a given vertex (e.g., words) being neighbors. As shown in Figure 6A, the *C* of the oral is higher than that of the written in both the interlanguage and the target language. The higher the *C* values for a vertex, the more aggregative the sub-network formed by the vertex and its neighbors. Put differently, the oral local sub-network in interlanguage is more aggregative than that in written language, and neighbors of a vertex in oral speech are also more likely to be connected. Additionally, the concentration in Chinese written and oral interlanguage is higher than that in the native Chinese system. Concerning the average path length, Figure 6B shows that the values for writing for Chinese native speakers exceed those for oral production, while the values of oral Chinese interlanguage networks exceed those of written interlanguage. The values of *L* for the eight interlanguage syntactic networks are within the range of 3.019–3.331. This suggests that the average distance between any two vertices in interlanguage syntactic networks is within three vertices. Liu (2008) has contended that the existence of the shortest path between any two vertices in a syntactic network is related to the minimization of the dependency distance. This refers to the linear distance between the governor and the dependent; the average dependency distance and the average path length are similar in some ways. Human languages tend to have a minimized average dependency distance, which is constrained by grammar and the human capacity for cognitive working memory. Thus, it can serve as a measure of processing as well as syntactic complexity (Hawkins, 2004). In linguistic syntactic networks, vertices represent word types. To some extent, the *L* between vertices can also reflect the involved working memory capacity for language processing. Previous studies have found no congruence between speaking and writing in terms of cognitive processes; the former includes conceptualization, modulation, and monitoring, while the latter is divided into planning, formulation, and monitoring. This means that language production processes and working memory related to speaking and writing differ (Levelt, 1989; Kellogg, 2001; Kuiken and Vedder, 2012). Oral production is generally considered to provide evidence of the implicit knowledge of a learner, whereas written production seems to allow for the use of explicit knowledge. Therefore, the cognitive load of writing is lower than that of speaking (Towell et al., 1996; Grabowski, 2007). Tasks involving vocal organs controlling, monitoring, and adjusting output compete for limited attention resources, and have trade-off effects in oral production. The oral task actually carries greater pressure to conceptualize the pre-verbal message for L2 learners because of the lack of accessibility for online planning (Skehan, 2009). According to the results, oral networks of CSL learners have longer *L*. This indicates that the working memory demands of CSL learners are higher when they complete oral tasks, whereas the working memory capacity required for writing tasks is slightly lower, and its average path length is also shorter. Previous studies have argued that L2 learners exhibit higher syntactic complexity in oral English and more lexical richness in writing. The findings are similar to those of Kormos and Trebits (2012), Zalbidea (2017), and Cho (2018). Additionally, Halliday (1989) focused on native language (L1), and also claimed that syntactic structures can be even more complex in speech than they are in writing.

The final value of *L* in L2 writing is closer to its oral production, while there is a significant difference in *L* between the oral and written language of native Chinese speakers. It has been suggested that the CSL learners are likely to “talk written down,” which has been revealed in previous studies of English interlanguage (Petch-Tyson, 1998; Cobb, 2003).

**Figure 7:**
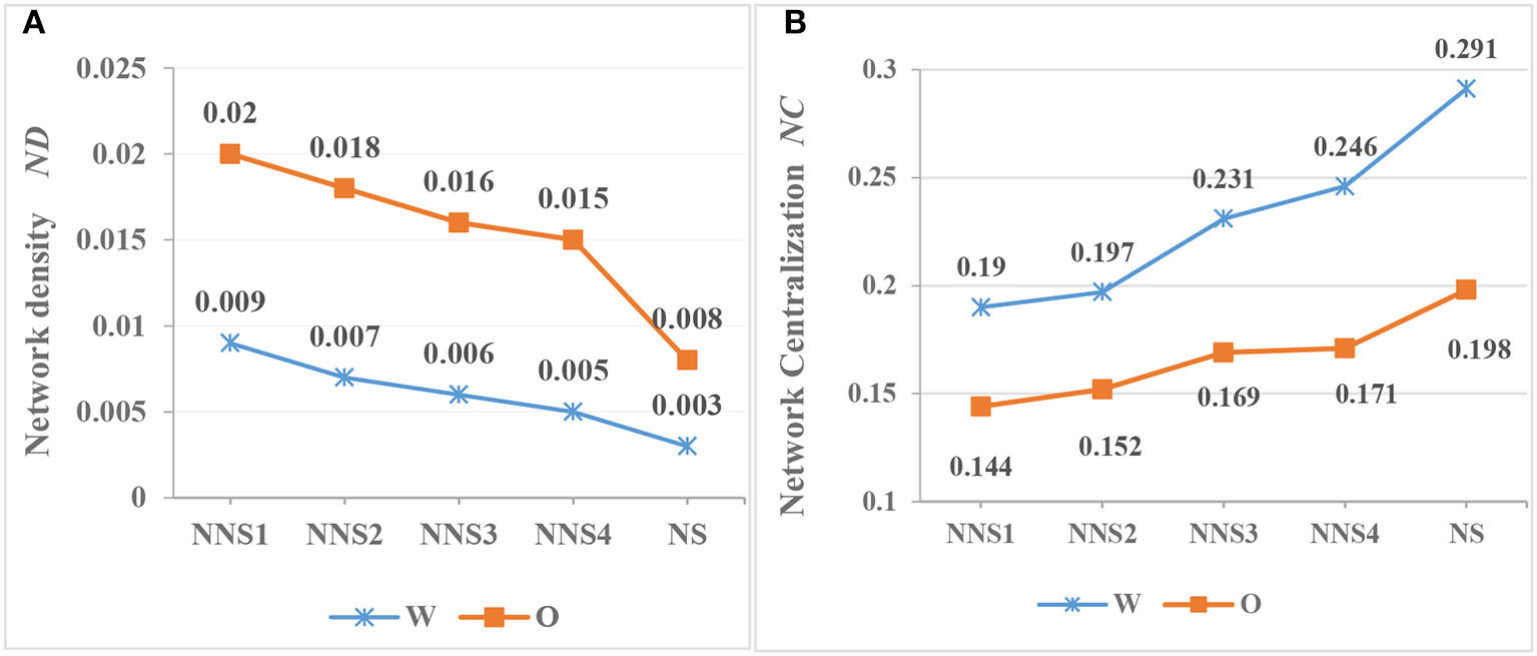
This is a figure with sub figures, **(A)** shows the values of *ND* in CSL learners' and native speakers' syntax networks, the values of *NC* in CSL learners' and native speakers' syntax networks are shown in **(B)**.

*ND* represents the correlation between vertices in the network, and *NC* reflects the difference between vertices and the likelihood there are hubs. As shown in Figure 7A, the *ND* of spoken interlanguage is always higher than that of written interlanguage. This indicates that the probability of syntactic dependency between two language units in spoken language is higher than that in written language. This indicates that the linear combination of language units in spoken language is more aggregative. It also shows that there are less vertices in spoken language than in written language. Moreover, a greater *NC* is respective of many hubs, and most of them are function words. According to Figure 7B, the changing trends for *ND* and *NC* are opposite. The *NC* values in written interlanguage are higher than those in oral language across four levels, and this shows that the combination ability of functional words in the writing of ECSL learners is stronger. Meanwhile, ECSL learners can produce richer vocabulary in writing than CSL learners. To summarize, CSL learners are likely to use cohesive devices more frequently in writing tasks.

### The Evaluation of Syntactic Network Properties on L2 Proficiency

Among the six complex network indicators investigated, five can differentiate the Chinese proficiency of learners; <*k*>, *C*, and *ND* are negatively correlated with Chinese proficiency, while γ′ and *NC* are positively correlated with Chinese proficiency.

The lower <*k*> is, the more vertices there are in a network, and the larger the vocabulary. The <*k*> of the interlanguage decreases with improvement in language proficiency, and approaches the target language. This indicates that ECSL learners acquire more vocabulary in both oral and written languages as Chinese input increases. Furthermore, in accordance with natural language networks, the probability distribution of the degree in interlanguage networks across the various levels and two modalities shows a decreasing trend, and only few vertices have extremely high degrees.

Regarding the power distribution of a scale-free network, because the value of its power exponent corresponding cumulative degree distribution parameter γ′ is closer to the value of 1, the more Zipfian distribution fits the data (Jiang et al., 2019). In this study, the γ′ of CSL learners does not approximate the value “1” as their Chinese proficiency improves. What is consistent with the findings of Jiang et al. (2019) is that the value of γ' is initially closer to 1. However, this does not indicate that L2 beginners have larger vocabularies than advanced L2 learners. Rather, because of the limited L2 input in the beginning stages of acquisition, ECSL learners are likely to repeat simple and similar words and structures. This results in a limited number of vertices with higher degrees. For example, CSL learners overuse the verb *you* (“have”), in both writing and speaking. ECSL learners use the verbs *you* and *zai* more than twice as often as native Chinese speakers in writing. Among the top five hubs, the frequency of CSL learners using *you* decreases from 20.25 to 15.28% as their proficiency improves; the frequency of *zai* drops from 14.3 to 10.85%. This means that L2 learners are less likely to produce basic syntactic structures.

The changes in the clustering coefficient in interlanguage decreases as levels of learners improve. Additionally, the probability that two vertices that are neighbors of a given vertex are neighbors of each other decreases. This is due to the increasing richness of vocabularies of learners. CSL learners use less limited language structures, increasing language structure difficulty and making their syntactic network sparse. These findings are consistent with Mellow (2006, 2008) and Jiang et al. (2019).

The smaller the *ND* value for a network, the thinner the edges of the network. Most language complex networks are sparsely distributed because the probability of syntactic dependencies between any two language units in a language subsystem is low (Liu, 2017). As indicated in Figure 7, as L2 proficiency improves, the network characteristics of the interlanguage approximate the natural target language network. This can also be attributed to the increasing vocabulary diluting the edges. However, CSL learners do not reach the level of TL at P4. This is consistent with Borodkin et al. (2016) who found that L2 lexical networks display greater local connectivity and less modular community structure than the network in the native language, even among proficient bilinguals. The constant increase in the *NC* indicates that the centrifugal and centripetal forces of hubs are improving, and the ability of CSL learners to use functional words increases. Chinese is not rich in morphological inflection, and its functional words are one of its most important syntactic devices. Thus, to an extent, the development of functional words can measure the interlanguage development of a learner (Skehan, 1998).

## Conclusions

Based on writing and oral treebanks of CSL learners, this study investigates the development of the syntactic networks of Chinese interlanguage with reference to the target language. We probe into the acquisition device of interlanguage and the influence of modalities and language proficiency on the development of interlanguage networks.

First, all interlanguage networks with different modalities present the scale-free and small-world properties in their initial stages. We confirm that there is no sudden syntactic emergence in interlanguage. From a macroscopic perspective of interlanguage, we hold that the regularity of L2 syntactic network development differs from that of L1 learners. This indicates that the role of the innate language acquisition device in L2 acquisition may be different from that of L1 acquisition. L2 acquisition is the process of interaction and connection between input and existing representation (Ellis, 2002). The existing native language knowledge is likely more important in L2 syntactic development than in the L1 acquisition of children.

Second, the values of quantitative parameters of interlanguage complex networks vary across modalities, and <*k*>, *L, C, ND*, and *NC* can differentiate the modalities. We found that the overall average degree in oral interlanguage is lower than that in written language. This indicates that lexical richness and the usage rate of functional words are more evident in writing than in speaking. Additionally, the average path is longer in the oral corpus than in the writing corpus, indicating that oral tasks require more working memory than written ones. As for the *ND* and *NC* of interlanguage, the two parameters for speaking and writing indicate more connection between the syntactic units in oral networks than among writing networks. Additionally, CSL writers usually aim to use more functional cohesive words and have a tendency to “talk written down.” This suggests that Chinese second language teachers should prioritize genre-based pedagogy.

Third, the interlanguage proficiency indicators for different modalities are the same, namely, <*k*>, γ′, *C, NC*, and *ND*, and each can be applicable as a syntactic indicator in measuring interlanguage development. We find that interlanguage is a non-linear and dynamic complex system that gradually approaches the target language. Although all of CSL learners' syntactic network's parameters approach the target language level with improvement in Chinese proficiency, they cannot achieve consistency with those of Chinese. This shows that the network of interlanguage is not as well-organized as that of the target language.

By virtue of the method commonly used in the complex network, we analyze the syntactic network development of Chinese interlanguage across modalities. We demonstrate that the syntactic development of interlanguage of L2 learners changes slower than that of their native language acquisition. Interlanguage is a self-organizing and self-regulated system under the influence of its native language knowledge. Some parameters in the complex network can differentiate modalities and language proficiency of learners. The construction and analysis of the interlanguage network based on authentic texts of L2 learners provide a new approach to the study of L2 acquisition. It also contributes to the infiltration of quantitative linguistics research methods into the field of L2 acquisition. However, this study does not investigate the similarities and differences in the development of the parameters of syntactic networks between native Chinese and second-language Chinese writing on the same topic of the writing and oral production. This may have influenced the results, and as such, should be controlled in future studies. At the same time, the corpora used in this study are collected from examination production, which deviates from the natural corpus of daily life. This likely influenced the objectivity of the research results. These conclusions could be further tested in future studies.

## Data Availability Statement

The original contributions presented in the study are included in the article/supplementary material, further inquiries can be directed to the corresponding author.

## Author Contributions

YH and HL conceived and designed the study. YH, XW, and MW collected the data and performed the statistical analysis. All the authors contributed in result interpretation and manuscript writing.

## Conflict of Interest

The authors declare that the research was conducted in the absence of any commercial or financial relationships that could be construed as a potential conflict of interest.

## Footnotes

^1^Pajek can be downloaded from http://mrvar.fdv.uni-lj.si/pajek/.
